# Editors' Note

**DOI:** 10.5195/ijt.2022.6469

**Published:** 2022-06-03

**Authors:** Ellen R. Cohn, Jana Cason

## INTERNATIONAL AUTHORSHIP AND READERSHIP

The International Journal of Telerehabilitation (IJT) is a biannual journal dedicated to advancing telerehabilitation by disseminating peer-reviewed information about current research and practices. This open-source journal is indexed by PubMed and Scopus. IJT enjoys a diverse international authorship and readership.

Such a diverse international audience would not be likely without the journal's use of an open journal system (OJS) and the generous sponsorship of the University of Pittsburgh's Office of Scholarly Communication and Publishing University Library System.

IJT does not actively engage in marketing. We surmise that this wide readership is testimony to the expanding global relevance of telemedicine, telehealth, and telerehabilitation. IJT requires no readership or author fees.

In the current issue, we are very pleased to publish work by authors from Australia, Belgium, Brazil, India, Israel, and Turkey, in addition to authors from the United States.

## ISSUE OVERVIEW

As context, this issue was produced during an historic time. COVID-19, a global pandemic is, even post-vaccine, still dramatically affecting healthcare worldwide to greater or lesser degrees, as well as the global economy. Submissions to IJT continue to increase, ostensibly to document how telerehabilitation is meeting those challenges. We regret that we are unable to publish the high volume of articles that are being submitted. Instead, we strive to promptly suggest other placements for work that is not central to telerehabilitation or relevant to the larger IJT audience.

The current issue of the multi-disciplinary *International Journal of Telerehabilitation* (IJT) features impactful research across rehabilitation disciplines including professionals engaged in cardiac rehabilitation; prevention, health, and wellness; occupational therapy; telerehabilitation post-organ transplantation; physical therapy (physiotherapy); and speech-language pathology. Some were generated by multi-disciplinary teams. Articles variously detail telerehabilitation across the lifespan.

## CALL FOR SUBMISSIONS

We cordially invite submissions to the Fall 2022 issue through October 31, 2022. IJT accepts original research, case studies, viewpoints, technology reviews, book reviews, and country reports that detail the status of telerehabilitation. References should adhere to the conventions of APA version 7.

Sincerely,

Ellen R. Cohn, PhD, CCC-SLP, ASHA-F

IJT Editor

Jana Cason, DHSc, OTR/L, FAOTA

Senior Associate Editor

